# Fe-Al-C carbide phase nano-layer investigation as a substrate for epitaxial diamond growth

**DOI:** 10.1186/s11671-015-0760-3

**Published:** 2015-02-05

**Authors:** Alexander Mekhed

**Affiliations:** G. V. Kurdyumov Institute for Metal Physics of the N.A.S. of Ukraine, 36 Academician Vernadsky Boulevard UA-03680, Kyiv -142, Ukraine

**Keywords:** K-phase, Diamond, Epitaxial growth, Electronic structure, Supercell, 81.10.Aj, 71.20.Be, 75.40.Mg

## Abstract

Calculations of electron structure of supercells consisting of several layers of ordinary stoichiometric K-phase and modified K-phase, on which according to our assumptions epitaxial growth of diamonds is possible, were conducted. Stability of calculated cells was considered, and optimal number of layers of the stoichiometric K-phase in the supercell was determined in order for it to be thermodynamically stable. Electronic structure of carbon in the modified K-phase layer was considered and compared to electron structure of carbon in diamond.

## Background

Fe_4−_*y*Al*y*C*x* (*у* = 0.4 to 1.0 and *х* = 0.08 to 0.66) known as K-phase is a carbide of Fe-Al-C system. Its following peculiarities can be marked out: its composition never reaches stoichiometry; depending on aluminum contents, it can be either paramagnetic or ferromagnetic; and its presence leads to formation of a martensite with anomaly high tetragonality. The presence of the K-phase in material has an impact on its significant properties [1]. But, probably, the most interesting peculiarity of the K-phase is its role as a diamond formation catalyst [2]. Despite the fact that conditions for diamond formation are more favorable in systems like Mn-Al-C and Ni-Mn-Al-C [3], the diamond formation in Fe-Al-C is still of an interest, e.g., from the point of view of creating grinding materials [4].

In our previous work, we have made an assumption that due to the proximity of crystal cell parameters, epitaxial growth of diamond on the K-phase is possible [5]. In the present work, the K-phase substrate will be considered, specifically, minimal necessary number of layers in order for that such structure to be stable. Electron structure of carbon atoms in substrate will be considered because similarities in electron structures may ease the diamond formation.

## Methods

WIEN2k was used in the work. It is a collection of computer programs that perform quantum mechanical calculations within the bounds of density functional theory using linearized plane waves method. This theory is based on the Kohn-Sham equation - a simplification of the Schrödinger equation in which electron system is described by the electron density functional. Many-electron system in this case is replaced with a system of noninteracting abstract particles which move in specific effective potential.

The stoichiometric K-phase was used as a subcell of a supercell because its use speeds up calculation while having little impact on final results. Unit cell parameter was equal to 0.375 nm multiplied by the number of subcells in a supercell.

Separation energy between valence and core states used in the calculations was equal to −7 Ry. The number of k-points in Brillouin zone was equal to 100 points per cell in all calculations. The following radii of atomic spheres were used: Al = 2.34 a.u., Fe = 1.87 a.u., and C = 1.66 a.u.

## Results and discussion

**Figure 1:**
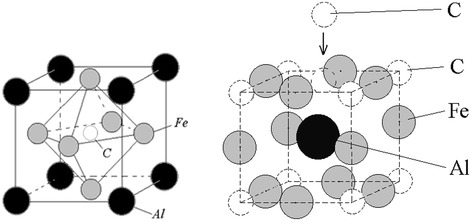
**Unit cells of K-phase.** Unit cells of K-phase: stoichiometric cell and modified unit cell of K-phase shifted along the vector [½ ½ ½].

Figure 1 shows a unit cell corresponding to the stoichiometric composition of the K-phase (on the left) and slightly modified unit cell (on the right) which was shifted towards the vector [½ ½ ½] (for clarity) and which has a carbon atom in place of octahedral pore. We assumed that this atom is deposited there during thermobaric treatment, and a layer containing this atom is the surface layer on which epitaxial growth of diamond is happening.

We have conducted an electron structure calculation of the K-phase supercells which consisted of several layers (one to three) of the ordinary stoichiometric K-phase and of one layer of modified unit cells (see description above). Table 1 contains enthalpies of formation of the supercells. As it can be seen, the supercells containing one to two intermediate layers have positive values of enthalpy. Thus to be formed, they need additional flow of heat. The supercells with three to four intermediate layers have negative values of enthalpy and thus can be formed without additional flow of heat. This means that supercells 3 to 4 have higher probability of formation compared to supercells 1 to 2.

**Table 1:** **Enthalpies of formation of the supercells consisting of several layers of stoichiometric K-phase and one layer of modified K-phase**

**Composition**	**Enthalpies of formation (eV/atom)**
One Fe_3_AlC + modified	0.017
Two Fe_3_AlC + modified	0.008
Three Fe_3_AlC + modified	−0.011
Four Fe_3_AlC + modified	−0.015

However, all these supercells have enthalpy of formation higher than that of Fe_3_AlC (−0.0318 eV/atom [5]) or Fe_3_AlC_0.5_ (−0.0335 eV/atom [5]). It means that they all need special conditions in order to be formed, e.g., presence of abundant carbon.

Values of Fermi energy decrease as the number of intermediate layers increase, which means that stability of the supercells also increase. In the presence of four intermediate layers, the Fermi energy value was equal to 0.81 Ry, which is comparable to the Fermi energy of the stoichiometric K-phase (0.77 Ry).

**Figure 2:**
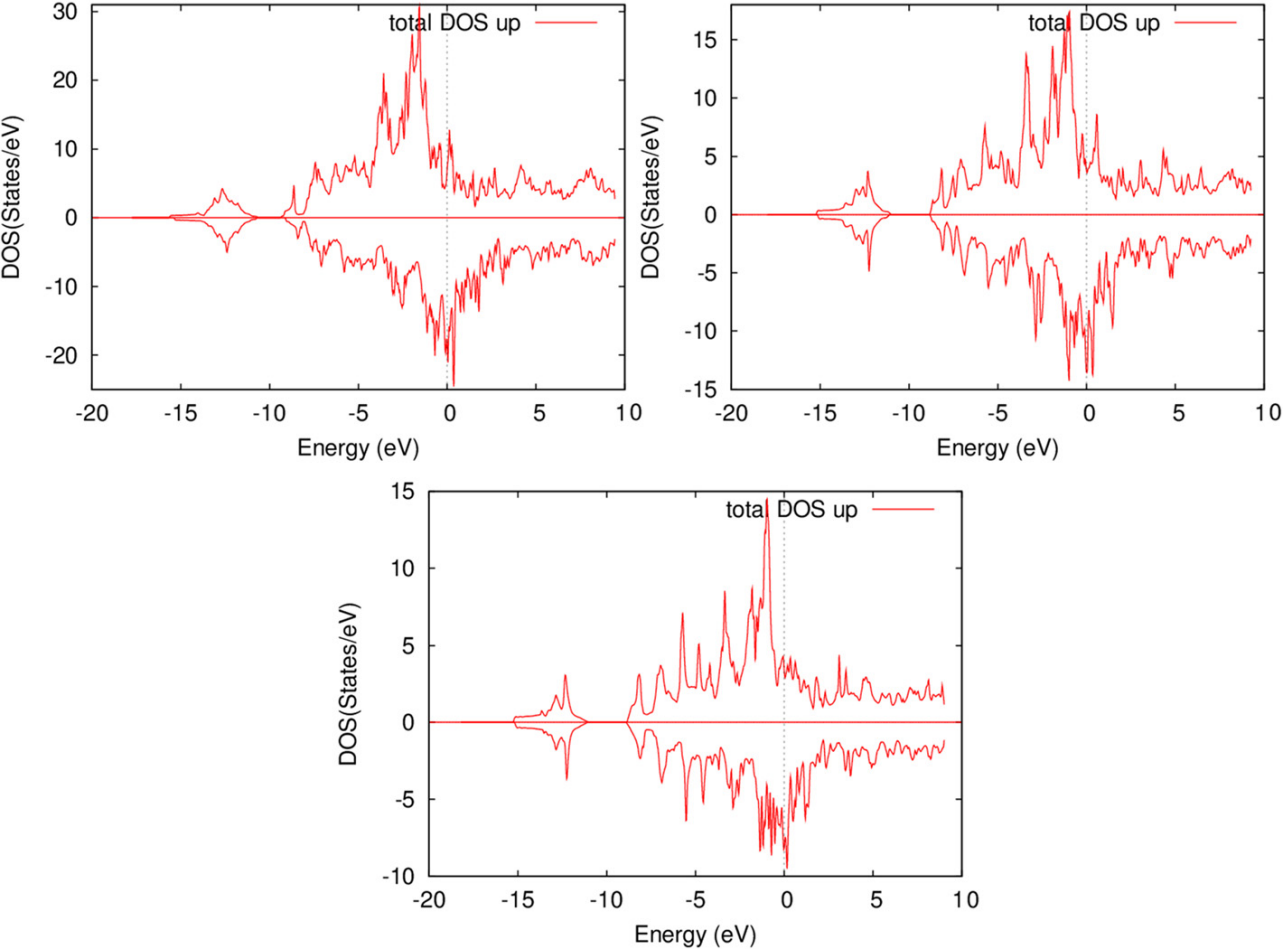
**Full densities of states of K-phase supercells.** Full densities of states of K-phase supercells with four, two, and one intermediate layers.

As in the stoichiometric K-phase, the Fermi level of electrons with spin-up falls on the minimum of pseudogap and for electrons with spin-down Fermi level falls on antibonding region (Figure 2) which results in a lower stability of the supercell. In contrast, the Fermi level of nonstoichiometric Fe_3_AlC_0.5_ and Fe_3_AlC_0.66_ cells falls on the minimum of pseudogap both for spin-up and spin-down electrons.

The minimum of pseudogap at the Fermi level of spin-down electrons in the supercells with one to two intermediate layers is indistinct. The Fermi level of the supercells with three to four layers does not fall on the minimum of densities of states, but the valence band peak is narrow. In general, all considered supercells are potentially unstable, same as Fe_3_AlC.

No substantial change in the densities of states happens with the increase of number of intermediate layers of the K-phase (Figure 2). Densities redistribute between local maximums, while position and distribution of maximums do not change. As a number of intermediate layers decrease, the densities of states redistribute closer to the Fermi level, which indicates of lower stability of such cells. As a number of layers increase, the distribution becomes more uniform.

On the basis of the above, one can state that the minimal number of intermediate layers of the K-phase is three to four since these cells have negative formation enthalpies and their electron structure has indications of stability.

Besides the proximity of unit cell parameters, another factor that makes the diamond formation on the K-phase easier is the similarities of electron structure of carbon atoms in diamond and substrate.

**Figure 3:**
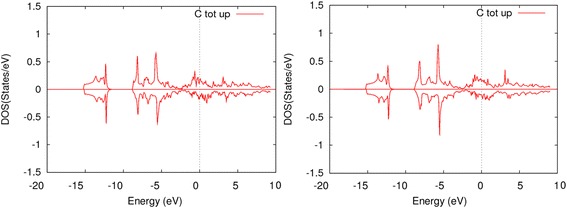
**Densities of states of carbon atom in modified K-phase layer.** Densities of states of carbon atom in modified K-phase layer supercell with three intermediate layers and with one intermediate layer.

The densities of states of carbon atoms in diamond are much more localized than that in the K-phase (Figure 3). Maximums (approximately −6 and −8.5 eV) of p-electrons are slightly (approximately 0.4 eV) shifted towards the Fermi level, but the distance between them are the same as in carbon atoms of diamond. S-electrons are significantly shifted towards the Fermi level. This is due to hybridization between s-states of carbon and d-states of iron. It is worth noting that there is no s-p hybridization in carbon atoms in the K-phase if the supercell contains only the K-phase layers, which can be attributed either to interaction with iron atoms or spatial disposition of the atoms. However, if the supercell contains a layer of diamond cells, hybridization in carbon atoms of the modified K-phase appears again.

There is also no gap between core and valence states in carbon atoms in the K-phase. This is due to overpopulation of lower energy levels in the supercells with electrons, and thus electrons start to occupy higher energy levels. However, one can see that the region, corresponding to gap in diamond, in the K-phase is underpopulated and its width is 3 to 5 eV, which corresponds to the width of the gap.

## Conclusions

Calculations of electronic structure of the K-phase supercells of Fe-Al-C system, which consisted of several layers of the stoichiometric K-phase and one layer of the modified K-phase with additional carbon atom, have shown that only supercells with at least three or four intermediate layers are thermodynamically stable. Supercells with smaller number of layers require additional flow of heat and are unstable.

On one side, the densities of states of carbon in the modified K-phase and diamond have similar features which might ease the diamond formation on the K-phase substrate. On the other side, due to iron influence, the carbon in the K-phase lacks s-p hybridization and s-states are shifted towards the higher energies.
